# Longitudinal Liver Stiffness Assessment in Patients with Chronic Hepatitis C Undergoing Antiviral Therapy

**DOI:** 10.1371/journal.pone.0047715

**Published:** 2012-10-17

**Authors:** Stella M. Martinez, Juliette Foucher, Jean-Marc Combis, Sophie Métivier, Maurizia Brunetto, Dominique Capron, Marc Bourlière, Jean-Pierre Bronowicki, Thong Dao, Marianne Maynard-Muet, Damien Lucidarme, Wassil Merrouche, Xavier Forns, Victor de Lédinghen

**Affiliations:** 1 Liver Unit, Hospital Clinic, IDIBAPS and CIBERehd, Barcelona, Spain; 2 Centre d’Investigation de la Fibrose hépatique, Hôpital Haut-Lévêque, CHU Bordeaux, Pessac, France; 3 Clinique Ambroise Paré, Toulouse, France; 4 Service d’hépato-gastro-entérologie, CHU Purpan, Toulouse, France; 5 Hepatology Unit, University Hospital of Pisa, Pisa, Italy; 6 Department of Hepato-Gastroenterology, Amiens University Hospital, Amiens, France; 7 Service d’hépato-gastroentérologie, Hôpital Saint-Joseph, Marseille, France; 8 Service d’hépatogastroentérologie, INSERM 954, CHU de Nancy, Vandoeuvre-les-Nancy, France; 9 Service d’Hépatogastroentérologie et de Nutrition, CHU Côte de Nacre, Caen, France; 10 Department of Gastroenterology and Hepatology, Hôpital de la Croix Rousse, Lyon, France; 11 Service de Pathologie Digestive, Université Nord de France, Groupe Hospitalier de l’Institut Catholique Lillois/Faculté Libre de Médecine Lille, Lille, France; The University of Hong Kong, Hong Kong

## Abstract

**Background/Aims:**

Liver stiffness (LS) measurement by means of transient elastography (TE) is accurate to predict fibrosis stage. The effect of antiviral treatment and virologic response on LS was assessed and compared with untreated patients with chronic hepatitis C (CHC).

**Methods:**

TE was performed at baseline, and at weeks 24, 48, and 72 in 515 patients with CHC.

**Results:**

323 treated (62.7%) and 192 untreated patients (37.3%) were assessed. LS experienced a significant decline in treated patients and remained stable in untreated patients at the end of study (*P*<0.0001). The decline was significant for patients with baseline LS ≥ 7.1 kPa (*P<*0.0001 and *P* 0.03, for LS ≥9.5 and ≥7.1 kPa *vs* lower values, respectively). Sustained virological responders and relapsers had a significant LS improvement whereas a trend was observed in nonresponders (mean percent change −16%, −10% and −2%, for SVR, RR and NR, respectively, *P* 0.03 for SVR *vs* NR). In multivariate analysis, high baseline LS (*P*<0.0001) and ALT levels, antiviral therapy and non-1 genotype were independent predictors of LS improvement.

**Conclusions:**

LS decreases during and after antiviral treatment in patients with CHC. The decrease is significant in sustained responders and relapsers (particularly in those with high baseline LS) and suggests an improvement in liver damage.

## Introduction

Liver fibrosis is a key determinant of morbidity and mortality in the natural history of CHC. There is evidence that antiviral therapy can improve liver histology not only by reversing liver damage in sustained responders, but also by slowing the progression in relapser patients. [1], [2].

Liver biopsy has been currently considered the reference standard to assess the extent of fibrosis, though it is associated with risk of complications and has limitations due to observer variability and sampling error.[3]–[5] Thus, several routine laboratory tests combined in scores and indices such as Forns’ score, APRI index and FIB-4 index, [6]–[9] or other panels like FibroTest (α2-macroglobulin, haptoglobin, apolipoprotein A1, gammaglutamyl transpeptidase and total bilirubin) [10] and more recently the ELF score (aminoterminal propeptide of type III procollagen (PIIINP), hyaluronic acid (HA) and tissue inhibitor of matrix metalloproteinase type 1 (TIMP-1)) [11] have been validated as useful tools to accurately detect significant fibrosis or cirrhosis in clinical practice. FibroTest, ELF score, Forns Score or other tests that include markers of extracellular matrix have been also validated in the evaluation of response to interferon-based therapy. [12]–[15].

More recently, transient elastography has emerged as a useful, rapid and reproducible tool to measure liver stiffness as an accurate marker to predict liver fibrosis degree.[16]–[20] Furthermore, the utility of elastography has also been evaluated in monitoring progression of fibrosis in the setting of hepatitis C virus recurrence after liver transplantation. [21].

In addition, changes in liver stiffness both during and after antiviral treatment have been previously examined by several other studies.[22]–[24].

The aims of this large prospective longitudinal multicentre study were to assess the effects of antiviral treatment and virologic response in liver stiffness and compare these changes with untreated patients with CHC. In addition, other biochemical and indirect tests of liver fibrosis were also assessed.

## Patients and Methods

### Ethics Statement

All patients provided written informed consent for blood samples and to data handling in accordance with a protocol specifically approved by the appropriate institutional review boards (IRB) which included: Hospital Clinic of Barcelona IRB and University Hospital of Bordeaux IRB for the centers in France.

### Study Population

From July 2008 through March 2009, we conducted this prospective multicentre study at ten participating sites in three European countries (Spain, France and Italy).

A total of 515 consecutive patients with CHC were enrolled in this study. The diagnosis of CHC was established by the presence of hepatitis C virus (HCV) RNA using polymerase chain reaction assays. Patients with human immunodeficiency virus or hepatitis B virus co-infection, or with other causes of chronic liver disease were not included.

### Transient Elastography

Liver stiffness measurement was performed using transient elastography (FibroScan, Echosens, Paris France) by the previously described technique. Briefly, with the patient lying in dorsal decubitus with the right arm at maximal abduction, a transducer probe on the axis of a vibrator is placed on the skin, between the rib bones at the level of the right lobe of the liver. Mild amplitude and low-frequency vibrations (50 Hz) are transmitted to the liver tissue, inducing an elastic shear wave that propagates through the underlying liver tissue. The operator in each center was a nurse who had previously performed at least 100 determinations in patients with chronic liver disease and who was unaware of patient´s status. Ten successful measurements were performed on each patient and the success rate was calculated as the number of validated measurements divided by the total number of measurements. The results were expressed in kilopascals (kPa). The median value of successful measurements was considered representative of the liver stiffness in a given patient, according to the manufacturer’s recommendations (interquartile range (IQR) less than 30% of the median value and success rate >60%). [25], [26].

### Serum Fibrosis Marker Panels

Blood samples were collected at baseline and during the study at weeks 24, 48 and 72. Laboratory tests included complete blood cell counts, HCV RNA serum concentration, HCV genotype, aspartate aminotransferase (AST), alanine aminotransferase (ALT), gamma glutamyl transpeptidase (GGT) and cholesterol. Marker panels of fibrosis including APRI and FIB-4 index were calculated as previously described.[7]–[9].

### Liver Histology

Indication of a liver biopsy was not mandatory in treated or untreated patients. It was offered to individuals as part of the evaluation for diagnosis and prognosis of the disease, in the setting of routine clinical practice in each center, independently of the final treatment decision. Percutaneous liver biopsies were performed under local anesthesia and ultrasound guidance with a Tru-Cut 14 gauge needle (Angiomed, Bard, Karlsruhe, Germany). Specimens were fixed in formalin, embedded in paraffin and stained with hematoxylin-eosin and Massońs trichrome. A minimum length of 10 mm and the presence of 6 portal tracts were required for diagnosis. Histological grade and stage were determined according to METAVIR scoring system [27] by a pathologist who was blinded for patients’ data. Liver fibrosis was considered significant when it spread out of the portal tract (stages 2, 3 or 4).

### Study Protocol

Treated patients included those who had stiffness values higher than 7.1 kPa (less likely to have absent or mild fibrosis according to previously suggested cut-off) [16] and those who wanted to receive antiviral treatment independent of their low liver stiffness values. Patients with stiffness values below 7.1 kPa or those who refused or had a contraindication for antiviral treatment remained untreated.

Liver stiffness measurements were obtained at baseline and at weeks 24, 48 (end of treatment) and 72 (end of follow-up) for G1-infected patients and at baseline and weeks 24 and 48 for G2/3-infected patients.

### Treatment

Antiviral treatment was the standard of care, with weekly pegylated interferon alfa-2a (180 ug) or alfa-2b (1.5 ug/kg) plus ribavirin (0.8–1.2 g daily) for 24 or 48 weeks, according to HCV genotype. The use of hematopoietic growth factors, epoetin alfa or darbepoetin and filgrastim, was allowed to treat anemia or neutropenia, respectively. Sustained virologic response (SVR) was defined by undetectable serum HCV RNA by qualitative polymerase chain reaction assay (Cobas Amplicor, HCV Roche, Branchburg, New Jersey, USA; v 2.0, detection limit 50 IU/mL) at 24 weeks after the end of therapy. According to stopping and futility rules, patients with a decrease of HCV RNA level <2 log_10_ IU at week 12 or a detectable HCV RNA at 24 weeks were considered to have treatment failure, and therapy was discontinued.

### Statistical Analysis

Descriptive values are expressed as percentages and the mean (±SD) or median (range). Quantitative data were compared using Student´s t-test or the non-parametric Mann–Whitney rank-sum test, as appropriate. The Chi-square test was used to evaluate categorical variables. The odds ratio, together with its 95% confidence interval (CI) and the corresponding P-value, was calculated for relative risks by using logistic regression. P values below 0.05 were considered statistically significant. The Wilcoxon matched pairs signed-rank test was used to evaluate changes between baseline and end of follow-up evaluations. To test for any associations with liver stiffness improvement, defined as a decrease of 20% or more from baseline LS values, variables with a P value of less than 0.1 on univariate testing were entered into a multivariate regression analysis. The Pearson’s correlation coefficient was used to analyse the correlations between values of liver elastography and ALT, FIB-4 index and APRI. The general linear model (GLM) for analyzing repeated measures technique was used to examine the changes of liver stiffness over time. All statistical analyses were performed with SPSS software (version 16.0, SPSS Inc, Chicago, IL).

## Results

Baseline clinical, laboratory and virologic characteristics of the patients are shown in Table 1. A total of 515 patients were evaluated: 323 treated patients (62.7%) and 192 untreated patients (37.3%). The mean age of the treated patients was 48.5 years, 66% were male and 56.7% were infected with HCV genotype 1. The mean age of untreated patients was 53.9 years, 35.9% were male and the vast majority (76.6%) were infected with HCV genotype 1. Treated patients had significantly higher baseline levels of serum ALT, AST and GGT, as well as higher histologic activity and fibrosis.

**Table 1:** Baseline characteristics of the patients.

*Variable*	Antiviral treatment cohort	Untreated	*P* value
	n = 323	n = 192	
Age (yrs)	48.5±11.2	53.9±11.7	<0.001
Sex (male)	214 (66.3)	69 (35.9)	<0.001
Body mass index (kg/m^2^ )	24.6±3.4	23.4±3.3	0.07
***AST/ULN***	1.9±1.4	1.1±0.6	<0.001
***ALT/ULN***	2.7±2.7	1.4±1.1	<0.001
***GGT/ULN***	1.5±1.4	1.2±1.1	0.001
Platelet count (10^3^/mm^3^)	206.6±67.6	239.6±55.9	<0.001
HCV RNA log_10_ (IU/mL)	5.8±0.9	5.8±0.8	0.5
HCV genotype			<0.001
1	186 (57.6)	147 (76.6)	
2	41 (12.7)	20 (10.4)	
3	76 (23.5)	9 (4.7.)	
4	17 (5.3)	13 (6.8)	
Other	3 (0.9)	3 (1.5)	
Fibrosis stage	**n = 189**	**n = 130**	<0.001
F 0–1	78 (41.3)	79 (60.8)	
F 2	60 (31.7)	41 (31.5)	
F 3	19 (10.1)	7 (5.4)	
F 4	32 (16.9)	3 (2.3)	
Histologic activity			0.05
A 0–1	123 (65.1)	97 (74.5)	
A 2	58 (30.7)	31 (24)	
A 3	8 (4.2)	2 (1.6)	

Results are expressed as the mean ± standard deviation or n (%).

ULN, upper limit of normal.

### Baseline Comparison of Liver Stiffness

Treated patients had significantly higher baseline LS than untreated patients (10.6±8.9 and 5.9±2.7, respectively, *P<*0.0001). Liver biopsies were carried out in 319 patients (189 patients in the treatment cohort). The stage of liver fibrosis was distributed as follows: F0, n = 45 (14.1%); F1, n = 112 (35.1%); F2, n = 101 (31.7%); F3, n = 26 (8.2%); F4, n = 35 (11%). The prevalence of significant fibrosis (F≥2) in this cohort was 50.9%. The areas under the receiver operating characteristic (ROC) curve of the FibroScan were 0.70 (95%CI, 0.62–0.74), 0.86 (95%CI, 0.81–0.92) and 0.87 (0.95%CI, 0.80–0.94), for F≥2, F≥3 and F = 4, respectively. Areas under ROC curve of APRI and FIB4 were 0.70 (95%CI, 0.63–0.75) and 0.65 (95%CI, 0.60–0.71), 0.78 (95% CI 0.72–0.85) and 0.70 (95%CI, 0.60–0.80), 0.80 (95%CI, 0.71–0.90) and 0.70 (95%CI,0.60–0.80), for F≥2, F≥3 and F = 4, respectively.

### Changes in Liver Stiffness during Treatment and According to Virologic Response

Mean liver stiffness values at each study time point for untreated and treated patients are shown in Table 2. After antiviral treatment, 202 patients (62.5%) achieved a sustained virologic response, while 121 patients (37.4%) did not. Among the latter, 66 patients (20.4%) had undetectable HCV-RNA at the end of treatment but then relapsed during follow-up. The mean interval between baseline elastography and end of study was 521.0±185.3 and 734.7±83.0 days for treated and untreated patients, respectively (*P*<0.0001).

**Table 2:** Liver stiffness variations during study and after follow-up and according to virologic response.

FibroScan (kPa)	Baseline	24 weeks	*P*	48weeks or EOT◊	*P*	72 weeks or 24 weeksof follow up◊	*P*
**Treated**	10.6±8.9♦	9.0±7.2	<0.001	8.8±7.0	<0.001	8.5±6.6	<0.001
**SVR**	9.3±5.9*	7.7±4.1	<0.001	7.7±4.7	<0.001	7.4±4.4	<0.001
** RR**	12.9±12.9*	11.4±9.9	0.009	10.9±9.5	0.01	10.1±8.7	<0.001
** NR**	12.4±11.3*	11±10.2	0.001	10.6±10.2	0.02	11.3±9.1	0.05
**Untreated**	5.9±2.7♦	6.3±3.4	0.3	6±3.3	0.8	6±3.2	0.7

Results are expressed as the mean.

* *P* 0.006 SVR vs RR and NR.

♦ *P*<0.0001.

◊ for untreated or treated patients, respectively.

**Figure 1:**
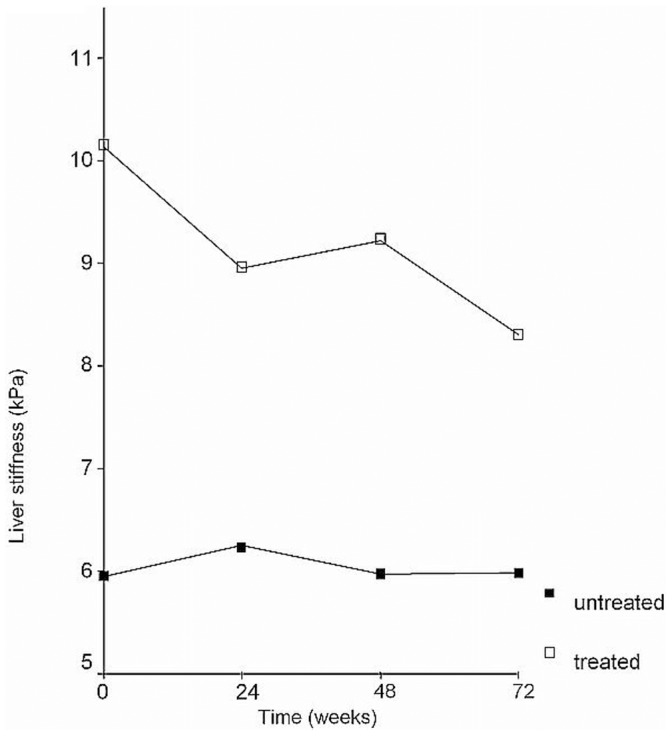
Liver stiffness evolution in treated *vs* untreated patients: Significant changes over time in treated *vs* untreated patients.

A significant decrease in LS values was observed only in treated patients whereas in untreated patients these measurements remained stable from basal assessment to the end of the study period (*P*<0.0001). The LS dynamic profile of treated versus untreated patients is shown in Figure 1, and is based on the GLM repeated measures analytical approach (*P*<0.0001).

**Figure 2:**
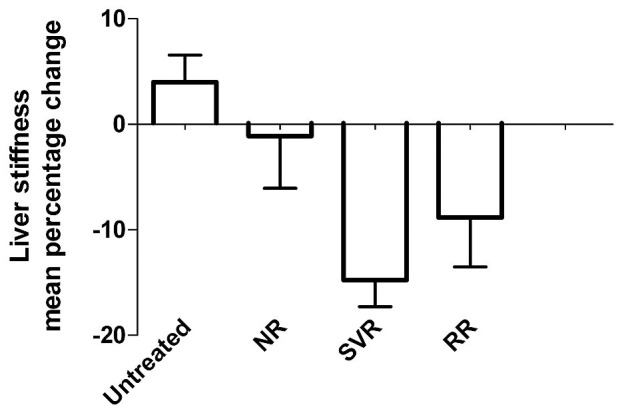
Mean percentage of change in liver stiffness from baseline to end of study according to treatment and virologic response.

The evolution of liver stiffness according to treatment and virologic response and the mean percentage of change over time in the 72-week period are shown in Table S1 (supporting material) and Figure 2, respectively. The dynamic profile according to virologic response is shown in Figure S1 (supplementary material). Compared with baseline, a significant reduction in liver stiffness was experienced by treated patients versus untreated (mean percentage change −12% *vs* 3%, *P*<0.0001). This decline was statistically significant for those patients with baseline LS ≥ 7.1 kPa versus those below this cut-off value (mean percent changes −22%, *P<*0.0001 and −18%, *P* 0.03, for baseline LS ≥9.5 kPa and ≥7.1 kPa, respectively). In the analysis according to the final virologic response, the baseline LS in sustained responders was significantly lower than in relapser responder and nonresponder patients (*P* 0.006). At week 24 and 48 all treated patients (sustained virological responders, relapsers and nonresponders) had significant LS decreases from baseline, with no different mean percentage changes between them. However, only sustained and relapser responders had a significant LS improvement at the end of study,(mean percentage change −16%, −10% and −2, for SVR, RR and NR, respectively, *P* 0.03 for SVR *vs* NR).

**Figure 3:**
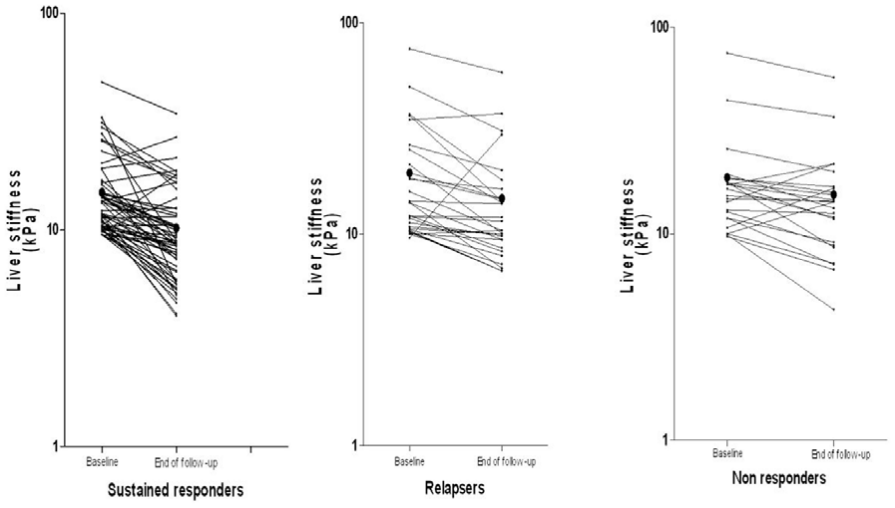
Liver stiffness evolution in patients with measurements above the cut-off value for advanced fibrosis and cirrhosis according to virologic response. The y axis is in logarithmic scale. The black dots indicate the mean liver stiffness value at each time points.

Among the 110 treated patients with baseline liver stiffness above the cut-off for advanced fibrosis and cirrhosis, values decreased below the cut-off level in 52 (47%) of them; interestingly the majority of them (70%) were sustained virological responders (Figure 3). The mean percent change in the sustained responders with LS values above cut-off for prediction of F3 (9.5 kPa) and F4 (12.5 kPa) was −25.5% and −30.8%, which resulted in a change to a lower stage of fibrosis in 80 and 60% of them, respectively.

ALT, AST and GGT serum values and FIB 4 index and APRI calculations had a significant correlation with LS at baseline (r = 0.33, 0.47, 0.34, 0.5, 0.6, respectively, *P* 0.0001). Similarly, ALT, FIB-4 index and APRI determinations demonstrated the evolution of LS according to treatment and virologic response, with significant differences at the end of study between SVR vs NR and RR (*P*<0.001). Serum ALT correlated significantly with LS in each time point of the study for each group of virologic response except for relapsers at 24 weeks post- therapy, where ALT (but not LS) showed a rebound (Figure S 1 B, supporting material).

### Predictors of Liver Stiffness Improvement

By univariate analysis, the following variables were associated with liver stiffness decline: male gender, low platelet count and time of follow-up, high body weight, body mass index (BMI), AST, ALT, GGT, histologic activity, baseline LS values, non-1 genotype, diabetes and antiviral treatment. In the final model of multivariate analysis, baseline higher LS values (odds ratio (OR) 1.14, 95% CI 1.0–1.2, *P*<0.0001), ALT levels (OR 1.0, 95%CI 1.001–1.009, *P = *0.01), antiviral therapy (OR 0.5, 95%CI 0.3–0.9, *P* = 0.003) and non-1 genotype (OR 1.06, 95%CI 0.4–1, *P* = 0.03) were independent predictors of LS improvement (Table 3).

**Table 3:** Factors associated with liver stiffness improvement.

	**Univariate analysis**			**Multivariate analysis**		
**Variables**	**Odds ratio**	**95% CI**	**P**	**Odds ratio**	**95% CI**	**P**
Male gender	1.54	0.08–2.7	0.01			
BMI≤25 Kg/m2	0.9	0.5–1.7	0.03			
Diabetes	0.6	0.2–2.2	0.001			
Genotype 1	0.6	0.4–0.9	0.004	0.6	0.4–1.0	0.03
Antiviral treatment	0.4	0.2–0.7	<0.001	0.5	0.3–0.8	0.003
Time between TE and end of FU	1	0.9–1	0.02			
Weight	1.02	0.9–1.0	0.01			
Platelet count	1	0.9–1	0.05			
AST	1	1.0–1.02	<0.001			
ALT	1.5	1.00–2.0	<0.001	1.005	1.0–1.01	0.01
GGT	1.0	0.8–1.2	0.001			
Liver stiffness	1.2	1.1–1.2	<0.001	1.14	1.0–1.2	<0.001
Histologic activity	1.6	0.9–2–8	0.08			

BMI, body mass index; FU, follow-up.

## Discussion

The primary aim of this study was to assess liver stiffness changes following treatment with pegylated interferon and ribavirin. The results demonstrate a significant stiffness decrease with antiviral treatment in comparison with untreated patients. According to the type of response, significant changes were detected only in sustained responders and relapsers. Previous studies had also shown a significant decrease in liver stiffness values in sustained responders.[22]–[24].

The improvement in liver stiffness at the end of study was particularly notable for those patients with higher pre-treatment liver stiffness values. As expected, two of the independent baseline predictors of the improvement were LS and ALT levels. The good correlation between LS and serum ALT levels during and after antiviral therapy, at least for sustained responders, as well as the association of LS improvement with ALT levels at baseline, is consistent with previous studies in which liver stiffness dynamic profiles ran in parallel with serum ALT in patients with CHC or in the course of acute hepatitis. [28], [29] Although ALT has some association with inflammatory activity in the liver, its association with variations in stiffness may reflect, to some extent, the influence of necroinflammatory changes on LS measurements, as was shown in the study by Fraquelli. [30] Moreover, according to univariate analysis, the histologic activity was also associated with stiffness improvement. The lack of correlation between liver stiffness and ALT at the follow-up measurement in the group of relapser patients might suggest that ALT changes are seen earlier than liver stiffness, which may more directly reflect necroinflammation/edema of reactivation once antiviral pressure is withdrawn.

The fact that nearly 50% of the patients with LS values above the cut-off for advanced fibrosis decreased to values of non-advanced fibrosis at follow-up may be in agreement with previous studies that demonstrate liver fibrosis regression by histological parameters in concordance with noninvasive tests assessment of fibrosis degree in patients receiving interferon-based therapy. [1], [2], [31] These results were even more striking in the subset of sustained virological responders, with a reduction of the predicted fibrosis stage in 80% and 60% of patients who had at baseline an estimated F3 and F4 according to the proposed cut-offs values, respectively. Indeed, the decrease in LS values in patients who achieved SVR were higher than 20% of baseline levels in a significant proportion of individuals with advanced liver fibrosis. The fact that stiffness decrease remained significant at follow-up only in sustained responders and relapsers may suggest its association with liver fibrosis regression.

Our study has some limitations. First, accuracy to diagnose significant fibrosis was lower than in other published reports. Since the study was not specifically designed to assess the performance of FS to evaluate liver fibrosis, the lower accuracy value may partially reflect “real-life” problems in diagnostic performance (liver biopsies were not evaluated by a single pathologist, biopsies not reaching a minimum desirable length might have been included). Nevertheless, accuracy to diagnose advance fibrosis and cirrhosis was excellent. A second limitation is that at any conclusions are necessarily constrained by the lack of available liver biopsies to definitively confirm their degree of regression at follow-up. Thus, based on our results and on previous observations, confounders other than liver fibrosis, mainly inflammatory activity, may partially influence these findings. A final limitation of our study is the short time of follow-up of patients, which may explain similar LS dynamics between sustained responders and relapsers, and no increase in LS values in untreated patients. Strengths of the current study were the large number of CHC patients enrolled from multiple centers who received the same combination antiviral therapy in a prospective study.

In summary, this study of a large cohort of patients with CHC confirms that a significant improvement in LS is associated with antiviral therapy in sustained responders and relapsers. Further evaluation of transient elastography in the long- term follow-up of changes in liver fibrosis in these patients is needed.

## Supporting Information

Figure S1 **Liver stiffness and serum ALT at weeks 24, 48 and 72, as compared with baseline, according to virologic response.** (A) Liver stiffness. (B) Serum ALT.
(TIF)

Table S1 **Liver stiffness, APRI, FIB-4 index and ALT evolution (mean delta change) from baseline to end of study.**
(DOC)

## References

[1] Poynard T, McHutchison J, Manns M, Trepo C, Lindsay K, et al (2002) Impact of pegylated interferon alfa-2b and ribavirin on liver fibrosis in patients with chronic hepatitis C. Gastroenterology. 122: 1303–13. 10.1053/gast.2002.33023 11984517

[2] Shiffman ML, Hofmann CM, Thompson EB, Ferreira-Gonzalez A, Contos MJ, et al (1997) Relationship between biochemical, virological, and histological response during interferon treatment of chronic hepatitis C. Hepatology. 26: 780–5. 10.1002/hep.510260335 9303513

[3] Perrault J, McGill DB, Ott BJ, Taylor WF (1978) Liver biopsy: complications in 1000 inpatients and outpatients. Gastroenterology 74: 103–6. 618417

[4] Regev A, Berho M, Jeffers LJ, Milikowski C, Molina EG, et al (2002) Sampling error and intraobserver variation in liver biopsy in patients with chronic HCV infection. Am J Gastroenterol 97: 2614–8. 12385448 10.1111/j.1572-0241.2002.06038.x

[5] Bedossa P, Dargère D, Paradis V (2003) Sampling variability of liver fibrosis in chronic hepatitis C. Hepatology. 38: 1449–57. 10.1016/j.hep.2003.09.022 14647056

[6] Forns X, Ampurdanès S, Llovet JM, Aponte J, Quintó L, et al (2002) Identification of chronic hepatitis C patients without hepatic fibrosis by a simple predictive model. Hepatology 36: 986–92. 12297848 10.1053/jhep.2002.36128

[7] Wai CT, Greenson JK, Fontana RJ, Kalbfleisch JD, Marrero JA, et al (2003) A simple noninvasive index can predict both significant fibrosis and cirrhosis in patients with chronic hepatitis C. Hepatology. 38: 518–26. 10.1053/jhep.2003.50346 12883497

[8] Sterling RK, Lissen E, Clumeck N, Sola R, Correa MC, et al (2006) Development of a simple noninvasive index to predict significant fibrosis in patients with HIV/HCV coinfection. Hepatology 43: 1317–1325. 16729309 10.1002/hep.21178

[9] Vallet-Pichard A, Mallet V, Nalpas B, Verkarre V, Nalpas A, et al (2007) FIB-4: an inexpensive and accurate marker of fibrosis in HCV infection. Comparison with liver biopsy and fibrotest. Hepatology 46: 32–6. 17567829 10.1002/hep.21669

[10] Imbert-Bismut F, Ratziu V, Pieroni L, Charlotte F, Benhamou Y, et al (2001) Biochemical markers of liver fibrosis in patients with hepatitis C virus infection: a prospective study. Lancet 357: 1069–75. 11297957 10.1016/S0140-6736(00)04258-6

[11] Rosenberg WM, Voelker M, Thiel R, Becka M, Burt A, et al (2004) Serum markers detect the presence of liver fibrosis: a cohort study. Gastroenterology 127: 1704–13. 15578508 10.1053/j.gastro.2004.08.052

[12] Poynard T, McHutchison J, Manns M, Myers RP, Albrecht J (2003) Biochemical surrogate markers of liver fibrosis and activity in a randomized trial of peginterferon alfa-2b and ribavirin. Hepatology 38: 481–92. 12883493 10.1053/jhep.2003.50319

[13] Martinez SM, Fernández-Varo G, González P, Sampson E, Bruguera M, et al (2011) Assessment of liver fibrosis before and after antiviral therapy by different serum marker panels in patients with chronic hepatitis C. Aliment Pharmacol Ther. 33: 138–48. 10.1111/j.1365-2036.2010.04500.x 21083589

[14] Fontana RJ, Bonkovsky HL, Naishadham D, Dienstag JL, Sterling RK, et al (2009) Serum fibrosis marker levels decrease after successful antiviral treatment in chronic hepatitis C patients with advanced fibrosis. Clin Gastroenterol Hepatol 7: 219–26. 19068241 10.1016/j.cgh.2008.10.034 PMC3766729

[15] Patel K, Benhamou Y, Yoshida EM, Kaita KD, Zeuzem S, et al (2009) An independent and prospective comparison of two commercial fibrosis marker panels (HCV FibroSURE and FIBROSpect II) during albinterferon alfa-2b combination therapy for chronic hepatitis C. J Viral Hepat. 16: 178–86. 10.1111/j.1365-2893.2008.01062.x 19175870

[16] Castéra L, Vergniol J, Foucher J, Le Bail B, Chanteloup E, et al (2005) Prospective comparison of transient elastography, Fibrotest, APRI, and liver biopsy for the assessment of fibrosis in chronic hepatitis C. Gastroenterology. 128: 343–50. 10.1053/j.gastro.2004.11.018 15685546

[17] Ziol M, Handra-Luca A, Kettaneh A, Christidis C, Mal F, et al (2005) Noninvasive assessment of liver fibrosis by measurement of stiffness in patients with chronic hepatitis C. Hepatology. 41: 48–54. 10.1002/hep.20506 15690481

[18] de Lédinghen V, Douvin C, Kettaneh A, Ziol M, Roulot D, et al (2006) Diagnosis of hepatic fibrosis and cirrhosis by transient elastography in HIV/hepatitis C virus-coinfected patients. J Acquir Immune Defic Syndr 41: 175–9. 16394849 10.1097/01.qai.0000194238.15831.c7

[19] Foucher J, Chanteloup E, Vergniol J, Castéra L, Le Bail B, et al (2006) Diagnosis of cirrhosis by transient elastography (FibroScan): a prospective study. Gut 55: 403–8. 16020491 10.1136/gut.2005.069153 PMC1856085

[20] Castera L, Forns X, Alberti A (2008) Non-invasive evaluation of liver fibrosis using transient elastography. J Hepatol 48 835–847. 18334275 10.1016/j.jhep.2008.02.008

[21] Carrión JA, Torres F, Crespo G, Miquel R, García-Valdecasas JC, et al (2010) Liver stiffness identifies two different patterns of fibrosis progression in patients with hepatitis C virus recurrence after liver transplantation. Hepatology 51: 23–34. 19839063 10.1002/hep.23240

[22] Ogawa E, Furusyo N, Toyoda K, Takeoka H, Maeda S, et al (2009) The longitudinal quantitative assessment by transient elastography of chronic hepatitis C patients treated with pegylated interferon alpha-2b and ribavirin. Antiviral Res 83: 127–34. 19443053 10.1016/j.antiviral.2009.04.002

[23] Wang JH, Changchien CS, Hung CH, Tung WC, Kee KM, et al (2010) Liver stiffness decrease after effective antiviral therapy in patients with chronic hepatitis C: Longitudinal study using FibroScan. J Gastroenterol Hepatol 25: 964–9. 20546451 10.1111/j.1440-1746.2009.06194.x

[24] Vergniol J, Foucher J, Castéra L, Bernard PH, Tournan R, et al (2009) Changes of non-invasive markers and FibroScan values during HCV treatment. J Viral Hepat 16: 132–40. 19175875 10.1111/j.1365-2893.2008.01055.x

[25] Sandrin L, Fourquet B, Hasquenoph JM, Yon S, Fournier C, et al (2003) Transient elastography: a new noninvasive method for assessment of hepatic fibrosis. Ultrasound Med Biol 29: 1705–13. 14698338 10.1016/j.ultrasmedbio.2003.07.001

[26] Lucidarme D, Foucher J, Le Bail B, Vergniol J, Castera L, et al (2009) Factors of accuracy of transient elastography (fibroscan) for the diagnosis of liver fibrosis in chronic hepatitis C. Hepatology. 49: 1083–9. 10.1002/hep.22748 19140221

[27] The French METAVIR Cooperative Study Group (1994) Intraobserver and interobserver variations in liver biopsy interpretation in patients with chronic hepatitis C. Hepatology. 20: 15–20. 8020885

[28] Coco B, Oliveri F, Maina AM, Ciccorossi P, Sacco R, et al (2007) Transient elastography: a new surrogate marker of liver fibrosis influenced by major changes of transaminases. J Viral Hepat 14: 360–9. 17439526 10.1111/j.1365-2893.2006.00811.x

[29] Arena U, Vizzutti F, Corti G, Ambu S, Stasi C, et al (2008) Acute viral hepatitis increases liver stiffness values measured by transient elastography. Hepatology 47: 380–4. 18095306 10.1002/hep.22007

[30] Fraquelli M, Rigamonti C, Casazza G, Conte D, Donato MF, et al (2007) Reproducibility of transient elastography in the evaluation of liver fibrosis in patients with chronic liver disease. Gut 56: 968–73. 17255218 10.1136/gut.2006.111302 PMC1994385

[31] Cammà C, Di Bona D, Schepis F, Heathcote EJ, Zeuzem S, et al (2004) Effect of peginterferon alfa-2a on liver histology in chronic hepatitis C: a meta-analysis of individual patient data. Hepatology 39: 333–42. 14767986 10.1002/hep.20073

