# Supplementary figures and images for: Longitudinal Liver Stiffness Assessment in Patients with Chronic Hepatitis C Undergoing Antiviral Therapy
Source: PLoS One. 2012 Oct 17;7(10):e47715. doi: 10.1371/journal.pone.0047715 (PMC3474716; doi:10.1371/journal.pone.0047715)

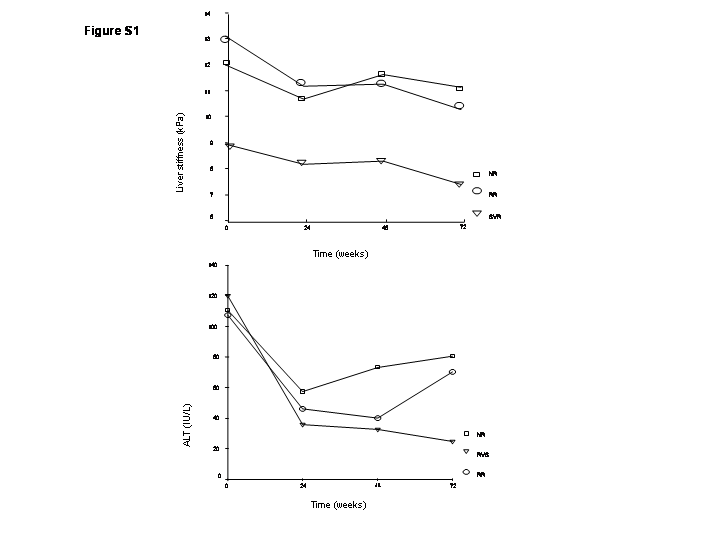

Supplement: Figure S1 — Liver stiffness and serum ALT at weeks 24, 48 and 72, as compared with baseline, according to virologic response. (A) Liver stiffness. (B) Serum ALT. (TIF) [file pone.0047715.s001.tif]
