# Supplementary material for: Longitudinal Liver Stiffness Assessment in Patients with Chronic Hepatitis C Undergoing Antiviral Therapy
Source: PLoS One. 2012 Oct 17;7(10):e47715. doi: 10.1371/journal.pone.0047715 (PMC3474716; doi:10.1371/journal.pone.0047715)
Supplement: Table S1 — Liver stiffness, APRI, FIB-4 index and ALT evolution (mean delta change) from baseline to end of study. (DOC) [file pone.0047715.s002.doc]

**Table S1. Liver stiffness, APRI, FIB-4 index and ALT evolution (mean** delta change) from baseline to end of study

|  | **Treated** | | | | **Untreated** |
| --- | --- | --- | --- | --- | --- |
|  | | | |
|  | **All (n:323)** | **SVR** | **RR** | **NR** |
| **FibroScan** | -1.9± 4.5* | -2±4 | -2.3±5.8 | -1.1±4.4 | 0.03±1.8* |
| **APRI** | -0.2±0.9* | -0.4±0.9^♦^ | 0.06 ±0.9^♦^ | -0.03±0.5^♦^ | 0.08±0.3* |
| **FIB-4** | -0.2±1.3* | -0.3±1^♦^ | 0.2±1.3^♦^ | -0.2±2^♦^ | 0.1 ±0.4* |
| **ALT** | -76.2±120.0* | -96±126^♦^ | -43.3±116^♦^ | -42±77^♦^ | -6.6 ±43.6* |

** P* <0.0001 treated *vs* untreated

♦*P <*0.001 SVR *vs* RR and NR
